# Complete chloroplast genome sequence of *Passiflora serrulata* Jacq. (Passifloraceae)

**DOI:** 10.1080/23802359.2020.1860711

**Published:** 2021-01-21

**Authors:** Hai-Fei Mou, Wei-Hua Huang, Jie-Yun Liu, Fang Wen, Qing-Lan Tian, Yan-Yan Wu, Long-Fei Fu, Ying-Jun Zhang, Yi-Gang Wei

**Affiliations:** a Biotechnology Research Institute, Guangxi Academy of Agricultural Sciences, Nanning, China; b Guangxi Key Laboratory of Plant Conservation and Restoration Ecology in Karst Terrain, Guangxi Institute of Botany, Guangxi Zhuang Autonomous Region and Chinese Academy of Sciences, Guilin, China

**Keywords:** Plastid genome, Passifloraceae, subg. Passiflora, phylogeny

## Abstract

This study was the first report for the complete chloroplast genome of *Passiflora serrulata* Jacq. (Passifloraceae). The cp genome was 149,683 bp in length contained two inverted repeats (IRs) of 25,470 bp, which were separated by large single-copy (LSC) and small single-copy (SSC) of 86,252 bp and 13,491 bp, respectively. A total of 110 functional genes were encoded, comprised 76 protein-coding genes, 30 tRNA genes, and four rRNA genes. The GC content was 37.0%. The maximum likelihood phylogenetic tree indicated that *P. serrulata* was recovered as the member of subg. *Passiflora* and most closely related to the clade formed by *P. serratodigitata* and *P. ligularis*.

*Passiflora* L. as the most species-rich genus in Passifloraceae comprises more than 600 species (Espinoza et al. 2018; Ma et al. 2019). The genus contains variable life forms such as lianas, vines, shrubs and small trees that distributed throughout southern and central America, southeastern Asia, Australia, and the Pacific Islands (Killip 1938; De Wilde 1972). Previous studies revealed that *Passiflora* contained five subgenera based on multiple loci from three genomes (Krosnick et al. 2009, 2013). This was incongruent with the phylogenies reconstructed by the dataset of 64 plastid encoded protein genes (Rabah et al. 2019; Mou et al. 2020). Furthermore, *Passiflora* exhibited highly accelerated rates of genomic rearrangements (Rabah et al. 2019) and nucleotide substitutions in plastid genomes (Shrestha et al. 2019). For further study on the phylogeny and plastid evolution of *Passiflora*, we reported the cp genome of *P. serrulata* for the first time.

In this study, leaves were collected from cultivated individual growing at Xishuangbanna Tropical Botanical Garden, Mengla County, Yunnan, China (N105°25′, E21°41′) and dried by silica gel for use in DNA extraction. Voucher specimen (MHF001) of this collection was deposited at herbarium of Guangxi Institute of Botany (IBK). Genomic DNA was extracted using CTAB method (Doyle and Doyle 1987) and then sent to Majorbio Company (http://www.majorbio.com/, China) for next generation sequencing. Short-insert (350 bp) paired-end read libraries preparation and 2 × 150 bp sequencing were performed on an Illumina (HiSeq4000) genome analyzer platform. Approximately 2 Gb of raw data was filtered using the FASTX-Toolkit to obtain high-quality clean data (http://hannonlab.cshl.edu/fastx_toolkit/download.html). The original data (SRR12846063) were mapped to the plastid genome reference (*Passiflora serratodigitata*, GenBank-MF807946) in Geneious Primer (Kearse et al. 2012) to exclude nuclear and mitochondrial reads. Putative chloroplast reads were then used for *de novo* assembling construction. Generated contigs were concatenated using the Repeat Finder function in Geneious Primer. The original data were repeatedly mapped to the larger contigs to extend their boundaries until only one contig remained. The IR region was determined using the Repeat Finder function in Geneious Primer and was reverse copied to obtain the complete chloroplast sequence. The annotation approach of cp genome of *Passilora serrulata* was performed followed Liu et al. (2018) using the same reference.

The complete chloroplast genome of *Passiflora serrulata* was 149,683 bp in length (GenBank-MT677873), the GC content was 37.0%. Large single-copy (LSC) and small single-copy (SSC) contained 85,252 bp and 13,491 bp respectively, while IR was 25,470 bp in length. The plastid genome encoded 110 functional genes, including 76 protein-coding genes, 30 tRNA genes, and four rRNA genes.

The maximum likelihood phylogenetic relationship was reconstructed by the dataset of 64 plastid encoded protein genes including 16 species of *Passiflora* as ingroup and one species of *Populus* as outgroup (Figure 1). The result was consistent with previous studies (Rabah et al. 2019; Mou et al. 2020) that subg. *Astrophea* and subg. *Decaloba* formed sister clade and together sister to subg. *Passiflora*. *Passiflora serrulata* was recovered as the member of subg. *Passiflora* and most closely related to the clade formed by *P. serratodigitata* and *P. ligularis*. The newly reported plastid genome will provide an addition for further study on the phylogeny and evolution of the genus *Passiflora* and of the family Passifloraceae.

**Figure 1.**
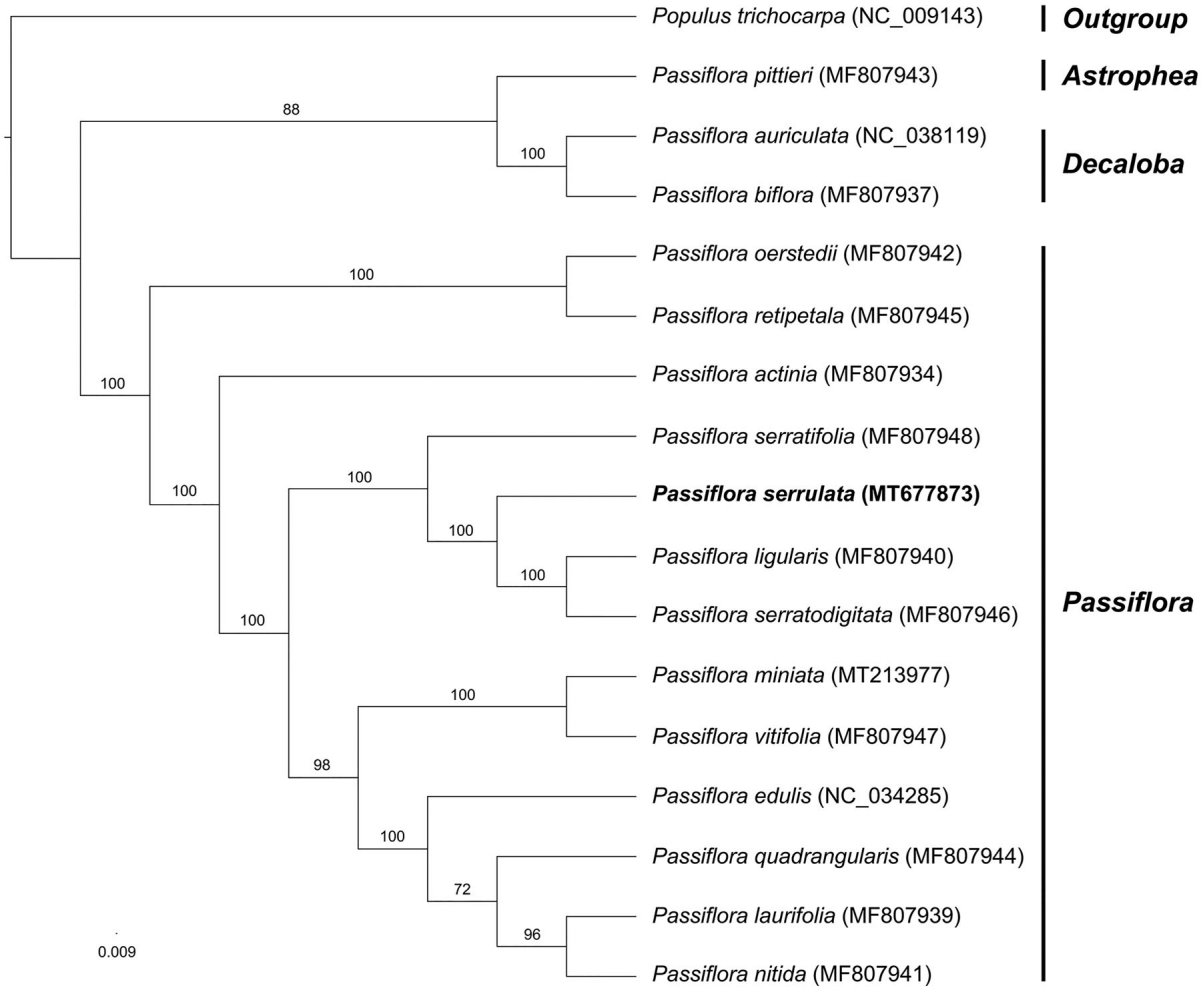
Phylogenetic tree reconstructed by Maximum Likelihood (ML) analysis based on 64 protein-encoding plastid genes, numbers upon branches are assessed by ML bootstrap.

## Data Availability

The genome sequence data that support the findings of this study are openly available in GenBank of NCBI at [https://www.ncbi.nlm.nih.gov](https://www.ncbi.nlm.nih.gov/) under the accession no. MT677873. The associated SRA number is SRR12846063.
